# Magnetic Resonance Image Feature Analysis under Deep Learning in Diagnosis of Neurological Rehabilitation in Patients with Cerebrovascular Diseases

**DOI:** 10.1155/2021/6051009

**Published:** 2021-10-25

**Authors:** Xue Li, Wenjun Ji, Hufei Chang, Chunyan Yang, Zhao Rong, Jun Hao

**Affiliations:** ^1^Department of Neurology, Yulin City No. 2 Hospital, Yulin, 719000, Shaanxi, China; ^2^Department of Neurosurgery, Yulin City No. 2 Hospital, Yulin, 719000, Shaanxi, China

## Abstract

To explore the impact of magnetic resonance imaging (MRI) image features based on deep learning algorithms on the neurological rehabilitation of patients with cerebrovascular diseases, eighty patients with acute cerebrovascular disease were selected as the research objects. According to whether the patients were with vascular cognitive impairment (VCI), they were divided into VCI group (34 cases) and non-VCI group (46 cases). In addition, based on the convolutional neural network (CNN), a new multimodal CNN image segmentation algorithm was proposed. The algorithm was applied to the segmentation of MRI images of patients with vascular cognitive impairment (VCI) and compared with the segmentation results of CNN and fully CNN (FCN). As a result, the segmentation results of the three different algorithms showed that the Dice coefficient, accuracy, and recall of the multimodal CNN algorithm were 0.78 ± 0.24, 0.81 ± 0.28, and 0.88 ± 0.32, respectively, which were significantly increased compared to those of other two algorithms (*P* < 0.05). The neurological evaluation results showed that the MMSE and MoCA scores of VCI patients were 15.4 and 14.6 ± 5.31, respectively, which were significantly lower than those of the non-VCI group (*P* < 0.05). The TMT-a and TMT-b scores of VCI patients were 221.7 and 385.9, respectively, which were significantly higher than those of the non-VCI group (*P* < 0.05). The FA and MD values of nerve function-related fibers shown in the MRI images of the VCI group were significantly different from those of the non-VCI group (*P* < 0.05). Therefore, the neurological recovery process of VCI patients was affected by multiple neurocognitive-related fiber structures. To sum up, the multimodal CNN algorithm can sensitively and accurately reflect the degree of neurological impairment in patients with cerebrovascular disease and can be applied to disease diagnosis and neurological evaluation of VCI patients.

## 1. Introduction

Cerebrovascular disease (VCD) is a common disease in the neurology department. Compared with other types of VCD, the mortality and disability rates are higher. Timely diagnosis and treatment after the onset of VCD is very important for the prevention of disability or death, which can improve or alleviate the pain of patients caused by the onset [1]. Vascular cognitive impairment (VCI) is a complex condition that ranges from mild neurocognitive impairment to dementia. It is caused by obvious or not obvious cerebrovascular diseases caused by cerebral ischemia and hypoxia [2]. As the onset of VCI is usually not detectable, the progression of the disease is slow and gradually aggravated with the progress of the disease, which tends to develop into vascular dementia over time. Therefore, if the optimal treatment time is missed, the recovery of neurological function will usually cause irreversible damage. Previous clinical trials found that some patients with VCD did not recover well after treatment, and VCI was common in the older population. With the increase of age, the prevalence of this disease increases, which is closely related to the lesions of cerebrovascular vessels. Clinical studies suggested that injuries and lesions in the left hemisphere and thalamus of human brain play a very important role in the impairment and recovery of neurocognitive function [3]. Most routine examinations of VCI are dominated by X-ray computed tomography (CT). CT can assist doctors to diagnose cases, but the use of this technology is limited, and the probability of misdiagnosis and missed diagnosis of patients is high [4]. In recent years, with the rapid development and extensive clinical application of medical imaging technology, magnetic resonance imaging (MRI) has become the main force in the analysis of medical imaging of cerebrovascular diseases. This diagnostic technique has greatly improved the clinical diagnosis rate of VCI and can accurately identify the location and nature of lesions, especially occupying a very important position in the diagnosis of some unique cases [5].

The traditional manual segmentation semantic model is particularly time-consuming and costly, and it is susceptible to human error because it relies on the rich clinical experience of the attending physician and the judgment of the subject. In recent years, with the development of science and technology such as big data and artificial intelligence, the application of deep learning algorithms, especially convolutional neural networks (CNN), has promoted the widespread application of image recognition and feature analysis techniques in the computer field in clinical medical diagnosis [6]. Meanwhile, CNN exhibits the unique characteristics of this algorithm and surpasses other technologies in natural image segmentation, which makes the exploration and development of cerebrovascular disease imaging diagnosis and feature recognition technology a good opportunity. The clinical results of medical experts also proved that MRI image diagnosis technology based on deep learning algorithm promotes the improvement of VCI clinical diagnosis rate in the diagnosis of cerebrovascular diseases [7].

Relevant studies found that compared with the traditional algorithm of MRI imaging technology, CNN-based MRI medical images can detect the brain nerve damage of patients earlier and more efficiently and can be further related to the degree of nerve damage in VCI patients [4]. Therefore, a new multimodal CNN image segmentation algorithm based on CNN was proposed. The algorithm was applied to the segmentation of MRI images of VCI patients and was compared with the segmentation results of CNN and FCN, to explore the effect of the algorithm on the neurological recovery of patients with different types of cerebrovascular diseases.

## 2. Materials and Methods

### 2.1. Research Objects and Grouping

From January 2019 to January 2020, 80 patients with acute cerebrovascular disease admitted to hospital were selected, with an age range of 68–81 years and an average age of 76 years. VCI was determined by referring to the criteria of vascular cognitive impairment without dementia used by the *Institute of Health and Aging* [8], and patients were divided into VCI group and non-VCI group. There were 34 patients in the VCI group, including 21 males and 13 females, with an average age of 77 years. There were 46 patients in the non-VCI group, including 29 males and 17 females, with an average age of 75 years. All enrolled patients underwent standardized procedures for medical history, neurological examination, and neuropsychological evaluation by an experienced neurologist. Laboratory examination and head MRI scan were performed, and diagnosis was made based on the above results. This study had been approved by the ethics committee of the hospital, and informed consent had been signed with patients and their families.

Inclusion criteria: (i) the age was no less than 55 years old; (ii) patients with at least one acute ischemic cerebrovascular disease had occurred for more than three months; (iii) patients conforming to the reference standards of cerebrovascular disease diagnostic criteria in *The Diagnostic Essentials of Various Cerebrovascular Diseases* revised by the *Fourth National Cerebrovascular Diseases Academic Conference of Chinese Medical Association* [9]; (iv) the informed consent of the subject and their family members was obtained.

Exclusion criteria: (i) patients with cortical and/or subcortical nonlacunar cerebral infarction and watershed infarction, intracerebral hemorrhage, normal cranial pressure hydrocephalus, and other white matter lesions; (ii) cognitive impairment for other reasons, such as normal cranial pressure hydrocephalus, hyperthyroidism or hypothyroidism, and vitamin B12 deficiency; (iii) patients with mental diseases, hemiplegia, aphasia, deafness, and patients who cannot cooperate to complete the psychological scale test.

### 2.2. Neurological Assessment

Neuropsychological tests and clinical interviews were conducted by neurologists trained with the neuropsychological scale on subjects and their families using standardized language. Detailed neuropsychological scales were tested in different cognitive domains. After the scale test, two evaluators scored independently, and the average value was taken. Evaluation scales included Montreal cognitive assessment (MoCA) Beijing version [10]. The neurocognitive functions specifically measured with tests including trail making test (TMT), clock drawing test (CDT), digit span test (DST), and verbal fluency test (VFT).

### 2.3. Imaging Examination

All subjects underwent craniocerebral scanning with a 3.0 T magnetic resonance imaging (GE SIGNA EXCITE II system) head coil. At the end of each scan, the original data were transmitted to the workstation through data transmission. All images were moved to eliminate image registration errors caused by head movement in scanning, and then FA images and MD images were generated by color 3D coding tensor. The FA and MD values of the splenium of corpus callosum (SCC), genu of the corpus callosum (GCC), fornix, pericallosal frontal (PCF), superior longitudinal fasciculus (SLF), and anterior cingulate field (ACF) were measured as indexes to evaluate the degree of white matter anisotropy and water molecular diffusion.

### 2.4. The Basic Structure of CNNs

Since its inception, CNN (CNN) has been widely used in tasks such as medical image classification, target recognition, and semantic segmentation, making it rapid in the field of medical imaging. However, the single use of CNN technology for medical image segmentation is not ideal. The segmentation technology has extremely high requirements for pixels, and different images will be used as detection objects, which put great demand on the segmentation technology. In response to the above problems, scholars such as Guo et al. [11] applied fully convolutional network (FCN) to medical image segmentation. The convolution process is as follows:

(1) $$\begin{array}{c} h_{j}^{x}=f\left(\underset{i\in M_{j}}{\sum }h_{j}^{x-1}p_{ij}^{x}+c_{j}^{x}\right). \end{array}$$

In equation (1), *p* is the convolution kernel, *x* is the number of layers, *p*_*ij*_^*x*^ is the weight, *c* is the deviation value, and *h*_*j*_^*x*−1^ is the output of the previous layer. The sampling expression in the pool layer is shown as follows:

(2) $$\begin{array}{c} h_{j}^{x}=f\left[u_{j}^{n}\text{down}\left(h_{j}^{x-1}\right)+c_{j}^{x}\right]. \end{array}$$

In equation (2), down(·) is the downsampling function, and *u* and *c* represent the multiplicative deviation and the additive deviation, respectively. This algorithm has an additional cropping layer and a multiloss structure, which can segment images of any size and solve the problem of gradient disappearance. The fully connected layer is expressed as follows:

(3) $$\begin{array}{c} h^{x}=f\left(t^{x}h^{x-1}+c^{x}\right). \end{array}$$

In equation (3), *t*^*x*^ is the weight coefficient, *h*^*x*−1^ is the output of the previous layer, and *c*^*x*^ is the bias term. Softmax can complete more than two classification tasks, and the expression is as follows:

(4) $$\begin{array}{c} J\left(\omega \right)=-\frac{1}{x}\left[\overset{x}{\underset{i=1}{\sum }}\overset{p}{\underset{j=1}{\sum }}1\left\{z^{i}=j\right\}\log\frac{e^{t_{j}^{m}s^{i}}}{\sum_{i=1}^{p}e^{t_{1}^{m}s^{i}}}\right]. \end{array}$$

In equation (4), *z*^*i*^ ∈ {1,2, ..., *P*} is the sample label, the total probability of normalized ∑_*j*=1_^*p*^*e*^*t*_*j*_^*m*^*s*^*i*^^ is 1, and the distribution in this algorithm is expressed as follows:

(5) $$\begin{array}{c} p\left(U=g|F\right)=\frac{e^{-L\left(g|F\right)}}{Z\left(F\right)}. \end{array}$$

In equation (5), *F* is the final output of FCN, *U*={*U*_1_, ..., *U*_*N*_} is the category label, and −*L*(*g|f*) is the energy function expression as follows:

(6) $$\begin{array}{c} -L\left(g|f\right)=\sum t_{i}\left(g_{i}\right)+\sum t_{ij}\left(g_{i},g_{j}\right). \end{array}$$

### 2.5. Segmentation Technology Based on Multimodal CNN

**Figure 1:**
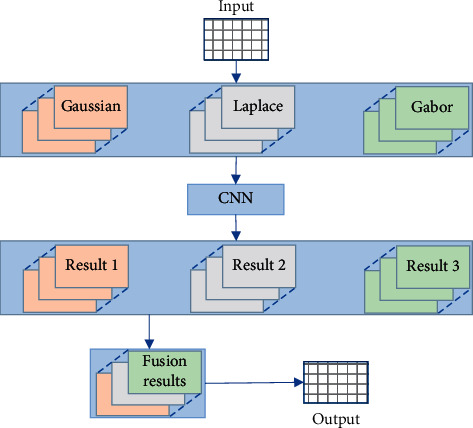
Multimodal CNN structure.

In Figure 1, the brain blood vessel segmentation of MRI images implemented was based on a deep learning network structure of multimodal CNNs. Through the input of the original image, multimodal processing was performed on the image first, including Gaussian, Laplacian, and Gabor filter processing. Then, it was segmented through the CNN, and the four modal results after the segmentation was merged. Finally, the fusion result was clustered based on Gaussian mixture model (GMM) and fuzzy C means (FCM) to get the final result. Such a network hierarchical structure realized the operation of semantic segmentation of cerebrovascular directly at the pixel level. The structure of the multimodal neural convolutional network is shown in Figure 1.

### 2.6. Evaluation Index of Cerebral Ischemia MRI Image Based on Deep Learning Algorithm

In the performance evaluation of tasks such as image classification and segmentation, the similarity (Dice coefficient), precision, and recall between the segmentation result and the standard segmentation were usually used to quantitatively describe the segmentation result [12]. The higher the Dice coefficient, the better the overall segmentation balance. The higher the recall rate, the more correct blood vessel points were segmented. The higher the correct rate, the greater the proportion of correct blood vessel points in the segmentation result. TP represents the correct rate of model prediction when the model prediction is true, TN represents the correct rate of model prediction when the model prediction is false, FP represents the false positive rate of model prediction when the model prediction is true, and FN represents the false miss rate of model prediction. The calculation equations for these three indicators were as follows.

(7) $$\begin{array}{c} \text{Dice}=\frac{2\text{TP}}{\text{FP}+\text{2TP}+\text{FN}}\text{,}\\ \text{precision}=\frac{\text{TP}}{\text{FP}+\text{TP}}\text{,}\\ \text{recall}=\frac{\text{TP}}{\text{FN}+\text{TP}}\text{.} \end{array}$$

### 2.7. Statistical Analysis

SPSS 24.0 was used for statistical analysis, and data were expressed as mean ± standard deviation ($\bar{\text{x}}$ ± *s*). One-way analysis of variance was used for intergroup comparison. Least significant difference (LSD) test was used when homogeneity of variance was satisfied, and *T*^2^ test was used when nonhomogeneity of variance was met. The test level was *α* = 0.05, and *P* < 0.05 was statistically significant.

## 3. Results

### 3.1. Comparison of the Effect of Three Algorithms on MRI Image Segmentation

**Figure 2:**
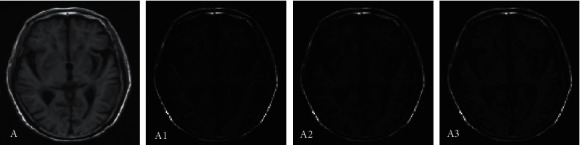
Comparison of MRI image segmentation at the top of the brain. Note: A represented the MRI image of the top of the brain; A1, A2, and A3 referred to the results of MRI image segmentation by CNN segmentation, FCN segmentation, and multimodal CNN, respectively.

Image segmentation was performed based on CNN, FCN, and multimodal CNN algorithms, and different segmentation results were obtained.  Figure 2 was the segmentation result of the MRI image of the top of the brain of a VCI patient. From the segmentation results of the three algorithms, the large blood vessels in the brain tissue can be segmented, and the difference between the different algorithms was mainly manifested in the cerebrospinal fluid. In addition, it was difficult for the three algorithms to segment small blood vessels in brain tissue, and the results of small blood vessel segmentation were not ideal. Of them, the segmentation effect of the CNN algorithm was the worst, and the segmentation effect of the multimodal CNN algorithm was the best, with the most of small blood vessels that can be segmented.

**Figure 3:**
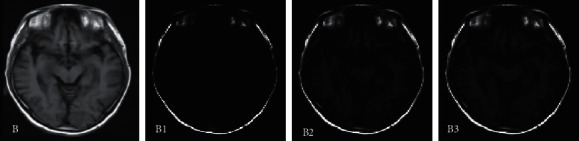
Comparison of MRI image segmentation in the middle of the brain. Note: B represented the MRI image of the middle of the brain, and B1, B2, and B3 referred to CNN segmentation, FCN segmentation, and multimodal CNN segmentation results, respectively.

Figure 3 was the segmentation result of the MRI image of the brain of a VCI patient. The segmentation results of the three algorithms were compared, and it was found that the large blood vessels in the brain tissue of the patient can be segmented. The difference between the different algorithms was mainly concentrated in the part close to the eye socket. Among them, the CNN algorithm had the worst effect on the skull segmentation, and there was a large error. The segmentation effect of the multimodal CNN algorithm was the best, the skull imaging was better, and the small blood vessels that can be segmented were the most.

**Figure 4:**
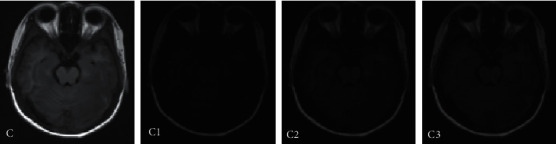
Comparison of MRI image segmentation at the bottom of the brain. Note: C represented the MRI image of the bottom of the brain, and C1, C2, and C3 referred to the results of MRI image segmentation by CNN segmentation, FCN segmentation, and multimodal CNN, respectively.

Figure 4 shows the segmentation result of the MRI image of the bottom of the brain of a VCI patient. From the comparison results of different algorithms, it was found that it was difficult for the three algorithms to segment the MRI image of the bottom of the brain. There were relatively many small cerebral blood vessels in the image, and the three algorithms had different degrees of segmentation errors. Among them, the segmentation quality of the multimodal CNN algorithm was relatively better, and it can segment more small blood vessels, followed by the FCN algorithm, and the error of the worst segmentation result of the CNN algorithm was relatively large.

### 3.2. MRI Image Segmentation Indicators of Three Algorithms

**Figure 5:**
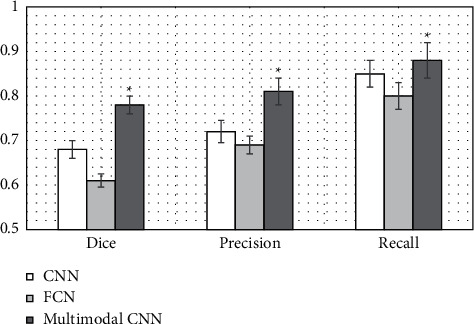
Comparison of the effect of three algorithms on MRI image segmentation. Note: ^∗^indicated that the three groups of analysis of variance had significant differences (*P* < 0.05).

Figure 5 shows the comparison result of the Dice coefficient, accuracy, and recall rate of the three algorithms segmented images. The comparative analysis results showed that the values of Dice coefficient, accuracy, and recall rate of the multimodal CNN algorithm were 0.78 ± 0.24, 0.81 ± 0.28, and 0.88 ± 0.32, respectively, which were considerably increased compared to those of the other two algorithms. The image effects of the three groups of algorithm segmentation had significant differences in Dice coefficient, accuracy, and recall rate (*P* < 0.05).

### 3.3. Comparison of the Scores of the Two Groups of Patients on the Neuropsychological Scale

#### 3.3.1. MoCA Score Results of the Two Groups of Patients

**Figure 6:**
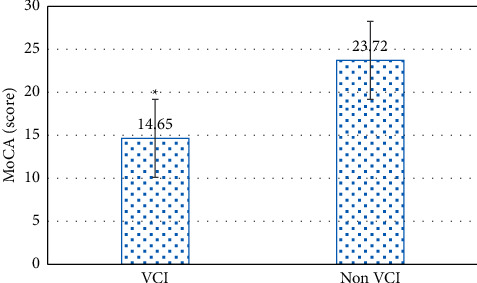
Comparison of MoCA scores between the two groups. Note: ^∗^indicated that compared with the non-VCI group, the MoCA scores of the two groups were considerably different (*P* < 0.05).

The MoCA scores of the two groups of cerebrovascular disease patients are shown in Figure 6. The results of the cognitive function scores of the VCI group and the non-VCI group showed that the MoCA scores of the VCI patients were 14.6 ± 5.31. Compared with the 23.7 ± 5.66 of the non-VCI group, the cognitive function scores of the VCI group were considerably reduced (*P* < 0.05).

#### 3.3.2. TMT-a and TMT-b Scores of the Two Groups of Patients

**Figure 7:**
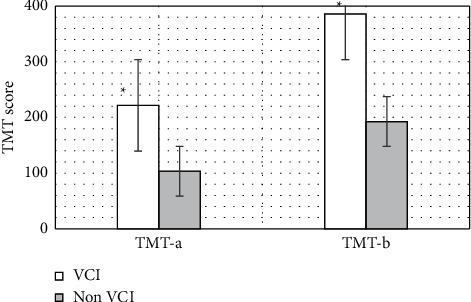
Comparison of executive function TMT scores between the two groups. Note: ^∗^indicated that compared with the non-VCI group, the scores of TMT-a and TMT-b between the two groups were considerably different (*P* < 0.05).

The executive function of TMT score results of the two groups of cerebrovascular disease patients is shown in Figure 7. The executive function score results of the VCI group and the non-VCI group showed that TMT-a and TMT-b scores of VCI patients were 221.7 and 385.9, respectively. Compared with the non-VCI group (103.3 and 192.7), the TMT scores of the VCI group were considerably increased (*P* < 0.05).

#### 3.3.3. CDT, DST, and VFT Score Results of the Two Groups of Patients

**Figure 8:**
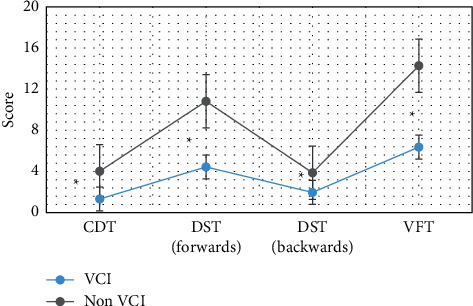
Comparison of CDT, DST, and VFT scores of the two groups of patients. Note: ^∗^indicated that compared with the non-VCI group, the CDT, DST, and VFT scores of the two groups were considerably different (*P* < 0.05).

The results of the trail making test (TMT), clock drawing test (CDT), digit span test (DST), and verbal fluency test (VFT) scores of the two groups of patients are shown in Figure 8. The CDT, DST (forwards), DST (backwards), and VFT scores of VCI patients were 1.32, 4.42, 1.96, and 6.37, respectively. Compared with the non-VCI group, the scores of the patients in the VCI group were considerably lower in these dimensions (*P* < 0.05).

### 3.4. Changes of FA Value of Neural Function-Related Fibers under Image Segmentation of Multimodal CNN Algorithm

**Figure 9:**
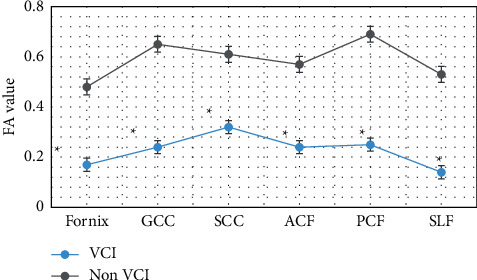
Changes in FA values of nerve function-related fibers in the two groups. Note: ^∗^indicated that compared with the non-VCI group, the FA values of the two groups were considerably different (*P* < 0.05).

Figure 9 shows the comparison of the changes in the FA values of nerve function-related fibers between the two groups. The results showed that the average FA values of Fornix, GCC, SCC, ACF, PCF, and SLF in the VCI group were 0.17, 0.24, 0.32, 0.24, 0.25, and 0.14, respectively. Compared with patients in the non-VCI group, the FA values of patients in the VCI group decreased considerably in each dimension, and the difference between the two groups was significant (*P* < 0.05).

### 3.5. Changes of MD Values of Neural Function-Related Fibers under Image Segmentation Based on Multimodal CNN Algorithm

**Figure 10:**
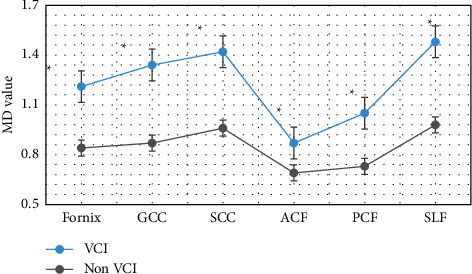
Changes in MD values of nerve function-related fibers in the two groups. Note: ^∗^indicated that compared with the non-VCI group, the MD values of the two groups were considerably different (*P* < 0.05).

Figure 10 shows the comparison of the changes in the MD values of nerve function-related fibers between the two groups of patients. The results showed that the average MD values of Fornix, GCC, SCC, ACF, PCF, and SLF in the VCI group were 1.21, 1.34, 1.42, 0.87, 1.05, and 1.48, respectively. Compared with patients in the non-VCI group, the MD values of patients in the VCI group increased considerably in each dimension, and the difference between the two groups was significant (*P* < 0.05).

## 4. Discussion

Cerebrovascular diseases that are not well diagnosed and treated often develop into VCI. The initial characteristics of VCI are not obvious, and the rate of medical diagnosis is not high, which leads to the rapid deterioration of the disease until the onset of dementia without good prevention and treatment. Therefore, good follow-up of patients with cerebrovascular diseases and their families and greater publicity are important ways to improve the diagnostic probability of VCI and prevent further dementia [13]. Many previous studies found that VCI is common in the elderly population, and its prevalence increases with the increase of age. This disease is closely related to cerebrovascular lesions. Clinical studies suggested that injuries and lesions in the left hemisphere and thalamus of human brain play a very important role in the damage and recovery of neurocognitive function [14]. Today, MRI has significant advantages in the examination and diagnosis of VCI. However, the actual clinical operation will be disturbed by subjective or objective factors such as the operating error of the attending physician, subjective expectation, and the patient's breathing rate. The errors caused by these disturbances will affect the final imaging quality of MRI, so it is particularly critical to integrate more appropriate algorithms into MRI technology [15]. For this reason, a new multimodal CNN-based MRI image segmentation algorithm was proposed and compared with the CNN algorithm and the FCN algorithm. The results showed that the Dice coefficient, accuracy, and recall of the multimodal CNN algorithm were 0.78 ± 0.24, 0.81 ± 0.28, and 0.88 ± 0.32, respectively. Compared with the other two algorithms, the image effects of the three groups of algorithm segmentation had significant differences in Dice coefficient, accuracy, and recall rate (*P* < 0.05). The research of Liu et al. [16] also found that the accuracy and sensitivity of multimodal CNN were significantly higher than those of traditional MRI. These data showed that the algorithm can automatically segment brain lesions with the smallest segmentation error and the highest segmentation accuracy.

Then, the image segmentation technology based on the multimodal CNN algorithm was applied to the MRI diagnosis of patients with cerebrovascular diseases, to explore its effect on the recovery of nerve function in patients with different types of cerebrovascular diseases. The comparison of the scores of the two groups of patients on the neuropsychological scale showed that the MoCA scores of VCI patients were 14.6, which were considerably inferior to those of the non-VCI group (*P* < 0.05). The TMT-a and TMT-b scores of VCI patients were 221.7 and 385.9, respectively, which were considerably superior to those of the non-VCI group (*P* < 0.05). Moreover, the CDT, DST (forwards), DST (backwards), and VFT scores of VCI patients were 1.32, 4.42, 1.96, and 6.37, respectively, which were considerably inferior to those of the non-VCI group (*P* < 0.05). This result was the same as that of Li et al. [17] on the correlation between cognitive impairment and MoCA score. A study by Farooq et al. [14] showed that the decline of executive function in patients with VCI was associated with deep white matter atrophy in the frontal lobe. In addition, the linear measurement indicators between different ventricles were significantly correlated with the scores of the subjects' neurocognition-related TMT, CDT, DST, VFT, and other domain cognitive scales. The above results all indicated that the cognitive function, which is mainly executive function, is completely impaired when ventricular anterior horn atrophy occurs, and its abnormal indicators can be used as a sign that the cognitive function of VCI is completely impaired.

In this study, FA and MD values of fiber bundles were measured on tomographic images, which did not require high MRI equipment conditions, and was easy to operate. Moreover, quantitative data of fiber bundle integrity can be obtained. Further statistical analysis was with more clinical value. The results of the study on the FA and MD values of fibers related to the recovery of nerve function showed that the FA values of patients in the VCI group were considerably reduced (*P* < 0.05), and the MD values related to each nerve fiber were considerably increased (*P* < 0.05). The research results of scholars such as Ye and Bai [18] also found that multimodal CNN can detect part of the brain nerve damage in a timely and efficient manner in VCI patients. In addition, it can be correlated with the degree of neurological damage in critically ill patients. These results indicated that VCI can damage multiple sets of cognitive-related fibers and affect fiber conduction function. Li et al. [19] also illustrated this point in the study on the influence of cerebrovascular diseases on neurological function. Therefore, as the severity of the disease progresses in VCI patients, the white matter fibrosis becomes more severe and the level of neurological function decreases.

## 5. Conclusion

Based on CNN, a new multimode CNN image segmentation algorithm was proposed. The algorithm was applied to the MRI image segmentation of VCI patients to explore its effect on the neurological recovery of patients with different types of cerebrovascular diseases. The results showed that the neurological recovery process of VCI patients was affected by multiple neurocognitive-related fiber structures. The multimodal CNN algorithm can reflect the degree of neurological impairment in patients with cerebrovascular disease sensitively and accurately and can be applied to the diagnosis and neurological function evaluation of patients with VCI. However, the sample size selected in this study is small, which is not convincing to some extent. Therefore, it should be considered to increase the sample size in the follow-up study to improve the research results.

## Data Availability

The data used to support the findings of this study are available from the corresponding author upon request.

## References

[1] Ferrer I., Vidal N. (2018). Neuropathology of cerebrovascular diseases. *Handbook of Clinical Neurology*.

[2] van der Flier W. M., Skoog I., Schneider J. A. (2018). Vascular cognitive impairment. *Nature Reviews Disease Primers*.

[3] Graff-Radford J. (2019). Vascular cognitive impairment. *Continuum: Lifelong Learning in Neurology*.

[4] Razek A. A. K. A., Elsebaie N. A. (2021). Imaging of vascular cognitive impairment. *Clinical Imaging*.

[5] Smith E. E., Beaudin A. E. (2018). New insights into cerebral small vessel disease and vascular cognitive impairment from MRI. *Current Opinion in Neurology*.

[6] Hamm C. A., Wang C. J., Savic L. J. (2019). Deep learning for liver tumor diagnosis part I: development of a convolutional neural network classifier for multi-phasic MRI. *European Radiology*.

[7] Carnevale L., Lembo G. (2019). Innovative MRI techniques in neuroimaging approaches for cerebrovascular diseases and vascular cognitive impairment. *International Journal of Molecular Sciences*.

[8] Hu M., Zhong Y., Xie S., Lv H., Lv Z. (2021). Fuzzy system based medical image processing for brain disease prediction. *Frontiers in Neuroscience*.

[9] Yan L., Zhou X., Zheng Y. (2019). Research progress in ultrasound use for the diagnosis and treatment of cerebrovascular diseases. *Clinics*.

[10] Li Y., Zhao J., Lv Z., Li J. (2021). Medical image fusion method by deep learning. *International Journal of Cognitive Computing in Engineering*.

[11] Guo X., Nie R., Cao J., Zhou D., Qian W. (2018). Fully convolutional network-based multifocus image fusion. *Neural Computation*.

[12] An F. P., Liu Z. W. (2019). Medical image segmentation algorithm based on feedback mechanism CNN. *Contrast Media & Molecular Imaging*.

[13] Barbay M., Taillia H., Nedelec-Ciceri C. (2017). Vascular cognitive impairment: advances and trends. *Revue Neurologique*.

[14] Farooq M. U., Min J., Goshgarian C., Gorelick P. B. (2017). Pharmacotherapy for vascular cognitive impairment. *CNS Drugs*.

[15] Stephen R., Liu Y., Liu Y. (2019). Brain volumes and cortical thickness on MRI in the Finnish geriatric intervention study to prevent cognitive impairment and disability (FINGER). *Alzheimer’s Research & Therapy*.

[16] Liu S., Yin L., Miao S., Ma J., Cong S., Hu S. (2020). Multimodal medical image fusion using rolling guidance filter with CNN and nuclear norm minimization. *Current medical imaging*.

[17] Li S., Shao Y., Li K. (2018). Vascular cognitive impairment and the gut microbiota. *Journal of Alzheimer’s Disease*.

[18] Ye Q., Bai F. (2018). Contribution of diffusion, perfusion and functional MRI to the disconnection hypothesis in subcortical vascular cognitive impairment. *Stroke and Vascular Neurology*.

[19] Li S., Lou X., Chang Z., Shi C., Lu H., Han J. (2020). Efficacy of neurointervention combined with intravenous thrombolysis in the treatment of ischemic cerebrovascular disease and its influence on neurological function and prognosis of patients. *Experimental and Therapeutic Medicine*.

